# Connexin Mutants Cause Cataracts Through Deposition of Apatite

**DOI:** 10.3389/fcell.2022.951231

**Published:** 2022-07-22

**Authors:** Peter J. Minogue, Andre J. Sommer, James C. Williams, Sharon B. Bledsoe, Eric C. Beyer, Viviana M. Berthoud

**Affiliations:** ^1^ Department of Pediatrics, University of Chicago, Chicago, IL, United States; ^2^ Molecular Microspectroscopy Laboratory, Department of Chemistry and Biochemistry, Miami University, Oxford, OH, United States; ^3^ Department of Anatomy, Cell Biology and Physiology, Indiana University School of Medicine, Indianapolis, IN, United States

**Keywords:** cataracts, mineralization, calcification, computed tomography, connexin, Fourier transform IR, gap junction channel, lens

## Abstract

Cataracts are lens opacities that are among the most common causes of blindness. It is commonly believed that cataracts develop through the accumulation of damage to lens proteins. However, recent evidence suggests that cataracts can result from calcium ion accumulation and the precipitation of calcium-containing salts. To test for the presence of precipitates and to identify their components, we studied the lenses of mice that develop cataracts due to mutations of connexin46 and connexin50. Micro-computed tomography showed the presence of radio-dense mineral in the mutant lenses, but not in wild-type lenses. Three-dimensional reconstructions of the scans showed that the distribution of the radio-dense mineral closely paralleled the location and morphology of the cataracts. The mutant lens homogenates also contained insoluble particles that stained with Alizarin red (a dye that stains Ca^2+^ deposits). Using attenuated total internal reflection micro–Fourier transform infrared spectroscopy, we identified the mineral as calcium phosphate in the form of apatite. Taken together, these data support the novel paradigm that cataracts are formed through pathological mineralization within the lens.

## Introduction

Cataracts are the leading cause of blindness worldwide (Resnikoff et al., 2004). They represent areas of cloudiness or opacification within the lens that prevent the proper transmission of light entering the eye onto the retina. Cataracts are frequently associated with aging and can develop as consequences of various diseases or in response to environmental insults. They can also result from genetic mutations of major lens proteins. The prevailing hypothesis has been that cataracts develop through accumulation of modifications in lens proteins (including oxidation, deamidation, cross-linking, cleavage, fragmentation, and glycation) and formation of insoluble high molecular weight protein aggregates (reviewed in Sharma and Santhoshkumar, 2009; Truscott and Friedrich, 2019).

The lens is an avascular organ comprised of an anterior epithelial cell layer and fiber cells that lose their organelles during differentiation. Because the lens has no direct blood supply, its homeostasis depends on a microcirculatory system that is responsible for the flow of water, ions and solutes into, within and out of the lens (reviewed in Mathias et al., 2007). In the circulation model, water and ions enter into the lens at the anterior and posterior poles; they flow through extracellular spaces into the lens center; and, they flow back to the surface and leave the lens through epithelial cells at the equator. This circulation is driven by ion pumps in the lens surface cells. As ions and water flow into the lens, they enter the cytoplasm of cells, following the electrochemical potentials of the ions, whereas outflow occurs by fiber cell-to-fiber cell diffusion through intercellular channels composed of the gap junction proteins, connexin46 (Cx46) and connexin50 (Cx50).

We have been characterizing two mouse cataract models that mimic connexin mutations found in humans, a Cx46 mutant (Cx46fs380) and a Cx50 mutant (Cx50D47A) (Berthoud et al., 2013; Berthoud et al., 2014; Gao et al., 2018; Berthoud et al., 2019). In heterozygous and homozygous lenses of both models, gap junction-mediated communication between lens fiber cells is significantly decreased (Minogue et al., 2017; Berthoud et al., 2019). The reduced intercellular communication impairs the lens microcirculation leading to disruptions of normal ion gradients, including that of calcium ions. The calcium ions accumulate to levels beyond the solubility product of some of its salts. After reaction of these lenses with Alizarin red (a dye used to detect calcified material in tissues like bone), we have observed red staining that closely corresponds to the locations of the opacities detected by darkfield microscopy (Gao et al., 2018; Berthoud et al., 2019). This led us to propose the novel hypothesis that cataracts result from accumulation of calcium ions and pathological mineralization within the organ by formation of deposits of calcium salts. Further support for our hypothesis that pathological mineralization is a common mechanism for cataract formation has been provided by studies of other mouse models in which Alizarin red-stained material is found in cataractous lenses (Li et al., 2021; Zhou et al., 2021).

The current study was designed to test the hypothesis that cataractous lenses expressing mutant lens fiber cell connexins contain mineralized material. Several approaches were applied to detect crystals or mineralized deposits within the connexin mutant lenses and to identify the inorganic components of these deposits. Part of these data was previously presented in abstract form (Beyer et al., 2019).

## Results

### The Cataractous Lenses Contain Radio-Dense Mineralized Material

**FIGURE 1:**
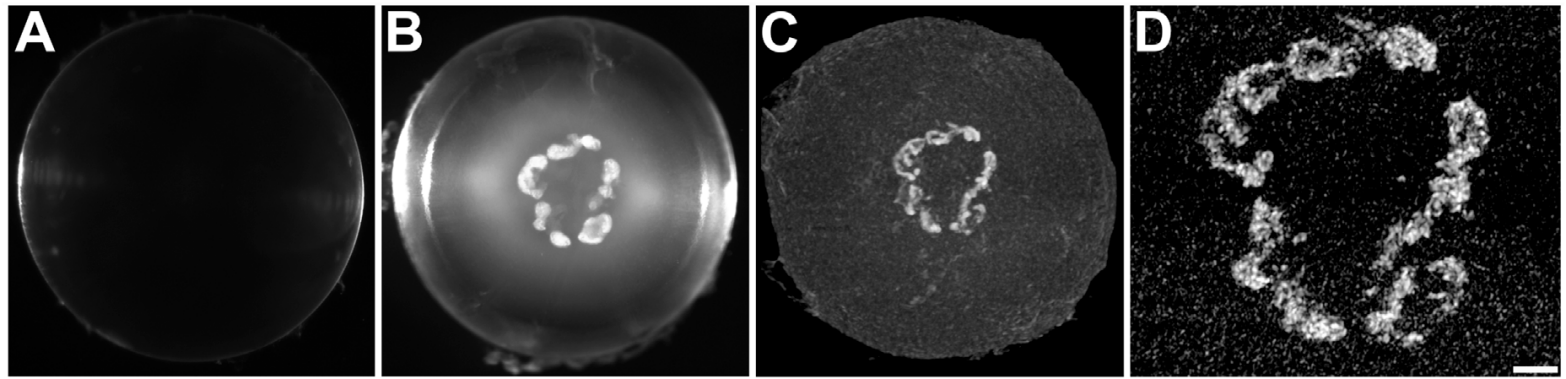
The distribution of the high X-ray attenuating material in Cx46fs380 lenses coincides with that of the cataract. **(A,B)** Darkfield images of Cx46fs380 lenses from 171-day-old wild-type **(A)** and homozygous **(B)** male mice. **(C)** Three-dimensional projection of the micro-CT images from the homozygous lens shown in **(B)**. **(D)** Magnified view of the central region of the three-dimensional projection of the homozygous lens shown in **(C)**. The scale bar represents 331 µm for panels **(A,B)**, 300 µm for panel **(C)**, and 100 µm for panel **(D)**.

**FIGURE 2:**
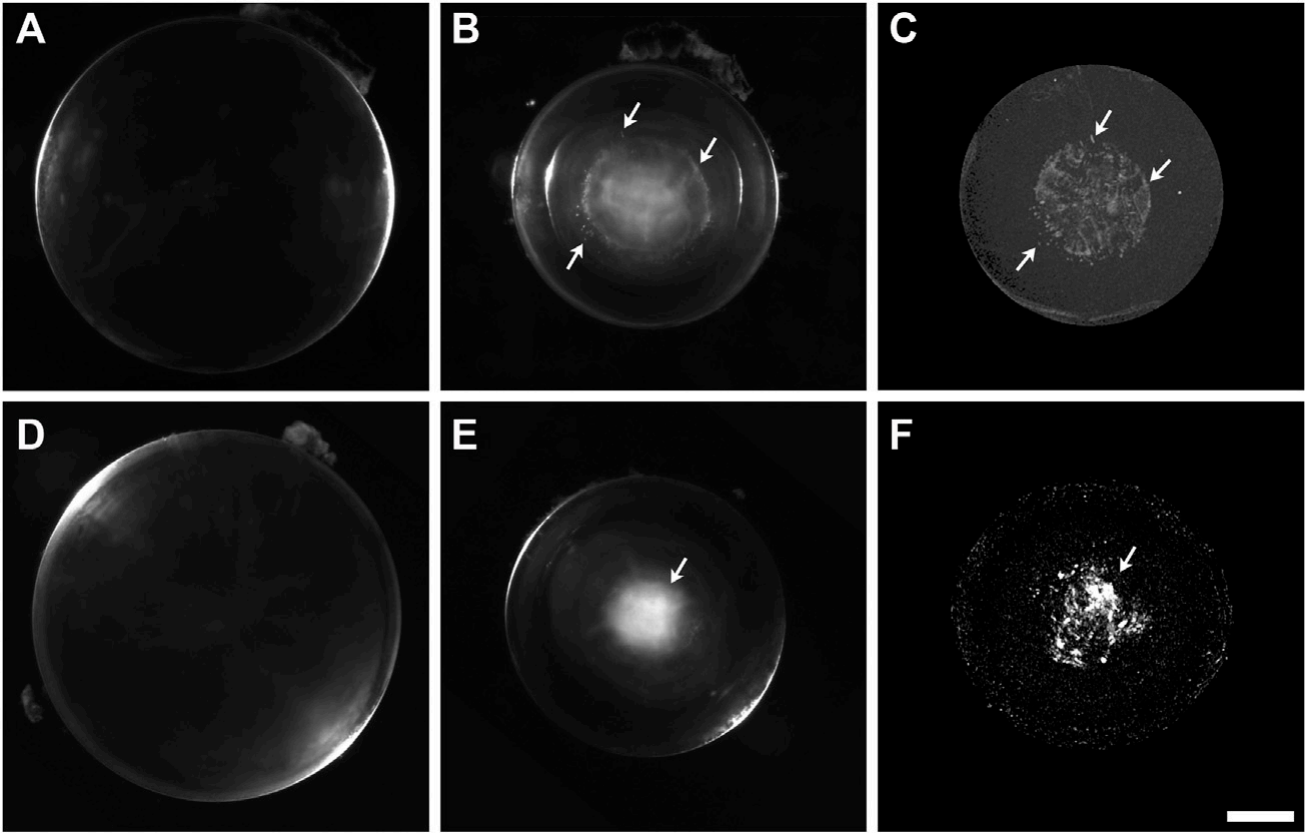
The cataractous Cx50D47A lenses present high X-ray attenuating material. **(A,B)** Darkfield images of Cx50D47A lenses from a 59-day-old wild-type **(A)** and a 56-day-old homozygous **(B)** male mouse. **(C)** View from a three-dimensional projection of the micro-CT images from the lens shown in **(B)**. A 3D-reconstruction movie of the mineral densities found in the lens shown in **(B)** is shown in Supplementary Movie S2. **(D,E)** Darkfield images of Cx50D47A lenses from 102-day-old wild-type **(D)** and homozygous **(E)** male mice. **(F)** View from a three-dimensional projection of the micro-CT images from the lens shown in **(E)**. A 3D-reconstruction movie of the mineral densities found in the lens shown in **(E)** is shown in Supplementary Movie S3. Arrows in panels **(B,C)** and **(E,F)** indicate positions of close correspondence between opacities seen in the darkfield images and X-ray dense material in the respective micro-CT images. The scale bar represents 357 µm for panels **(A,B)**, 418 µm for panel **(C)**, 448 µm for panels **(D,E)**, and 378 µm for panel **(F)**.

We used high energy, micro-computed tomography (micro-CT) scanning to examine the lenses for the presence and distribution of mineralized material. We studied homozygous animals, because they have more severe cataracts than the heterozygotes. The Cx46fs380 animals that were studied were older than the Cx50D47A, because they develop severe cataracts later. We obtained darkfield microscopy images of freshly dissected wild-type and homozygous Cx46fs380 and Cx50D47A lenses. Then, the lenses were fixed and studied by micro-CT scanning. The wild-type lenses appeared transparent by darkfield microscopy and showed no radio-dense material in the micro-CT images (Figures 1A, 2A). In contrast, the homozygous mutant lenses had cataracts (Figures 1B, 2B,E), and they contained abundant radio-dense material (Figures 1C,D, 2C,F). Although the examples shown are from male mice, X-ray dense material was observed in mice of both sexes.

Cataracts in the homozygous Cx46fs380 lenses were composed mostly of several large opacities within the anterior region of the lens nucleus (Figure 1). Three-dimensional reconstruction of the micro-CT scans of the lenses from 5-6-month-old homozygous Cx46fs380 mice showed relatively large mineral bodies concentrated in a plane corresponding to the anterior region of the lens nucleus and scattered bits of mineral spread out towards the posterior pole of the lens (Figure 1; Supplementary Movie S1). In the lens shown in Figure 1, the mineral bodies were arranged roughly into a flattened ring that encircled the anterior region of the lens. The distribution of the mineral particles (detected by micro-CT) corresponded nearly perfectly to the morphology of the cataracts (seen by darkfield microscopy) (Figure 1). In Cx46fs380 lenses, the length of the mineral bodies ranged from 73–2,936 μm and the width ranged from 41–240 μm (*n* = 7). Consistent with the appearance and restricted location of the Cx46fs380 cataracts, the proportion of the lens volume that contained mineral as determined from the micro-CT data was 0.31% (range 0.09%–1.03%; *n* = 7).

The distribution of mineral and the appearance of the radio-dense deposits in homozygous Cx50D47A lenses were very different from the Cx46fs380 lenses. Homozygous Cx50D47A animals showed a rather severe cataract that optically appeared diffuse and occupied the entire lens nucleus (Figure 2). While wild-type lenses showed no X-ray dense material in any region, Cx50D47A lenses showed widely dispersed particles with a dim X-ray density and some particles that were more intensely X-ray dense. The more X-ray dense particles extended into curved, needle-like shapes or coalesced into thin, spherical shells. On average, the X-ray dense material in the Cx50D47A lenses occupied only 0.24% of the lens volume (range 0.08%–0.51%; *n* = 6) in lenses from 2-month-old mice and 0.84% (range 0.45%–1.33%; *n* = 8) in 102-day-old lenses. The total X-ray dense material (a combination of scattered particles of dimly X-ray dense material extending up to the center of the lens nucleus in between thin, spherical baskets of higher X-ray density) occupied a spherical volume that closely matched the region of the cataract in each lens (Figure 2; Supplementary Movies S2, S3). The elongated appearance of some of the X-ray dense regions resembled the shapes and orientations of fiber cells. The lengths of highly X-ray dense deposits in the spherical layers ranged from 58–458 μm (*n* = 6) in 2-month-old lenses and 160–348 μm (*n* = 8) in 102-day-old lenses, and the widths ranged from 8–65 μm (*n* = 6) and 21–61 μm (*n* = 8), respectively, suggesting that their dimensions increased with age (as expected, because the cataracts become more severe with age). Interestingly, the average widths of the mineral layers were significantly thinner than the average widths of the mineral bodies in the Cx46fs380 lenses (*p* < 0.02).

### Cataractous Lenses Have Insoluble Particles That Stain With Alizarin Red

**FIGURE 3:**
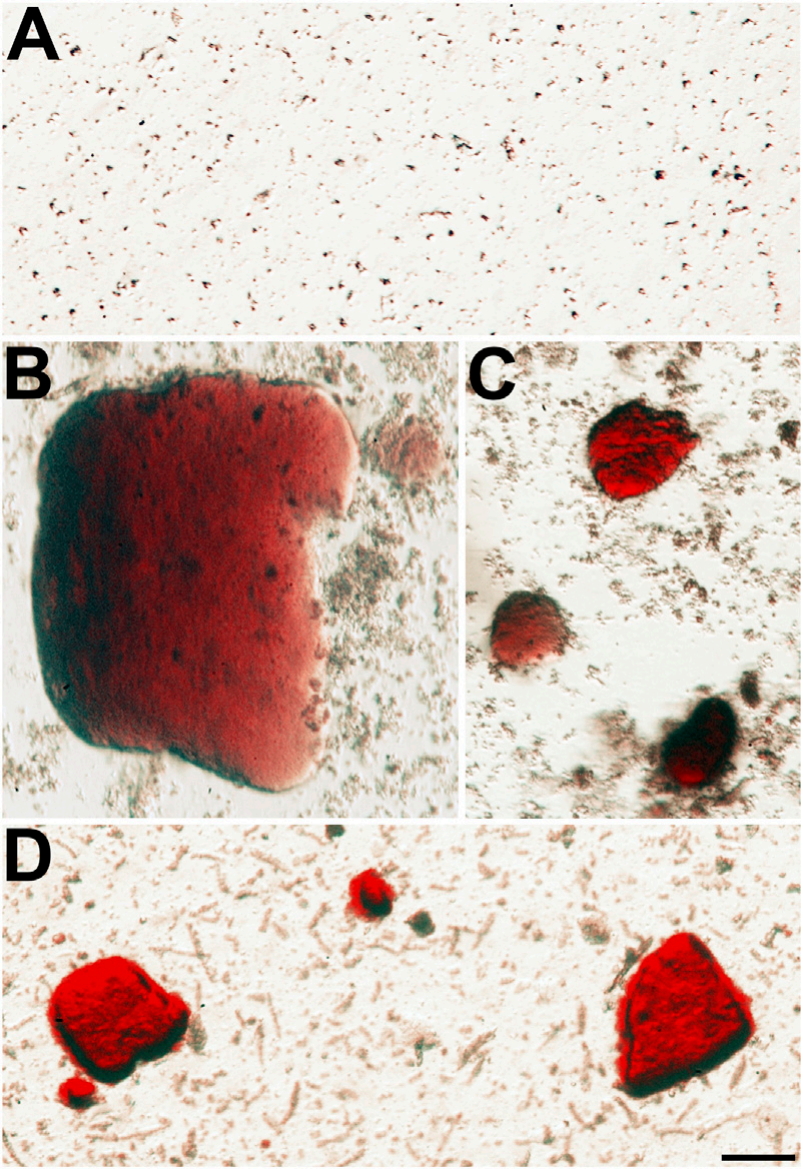
Alizarin red-stained crystals are present in the insoluble fraction of homozygous Cx46fs380 lenses. **(A)** Image shows the absence of Alizarin red-stained material in the lens insoluble fraction of a 191-day-old wild-type Cx46fs380 male mouse. **(B−D)** Images show the presence of Alizarin red-stained crystals of different sizes in the lens insoluble fraction of a homozygous Cx46fs380 female littermate. The images were modified to decrease the reddish background from the Alizarin red solution in which the insoluble fraction was resuspended. The scale bar represents 54 µm.

**FIGURE 4:**
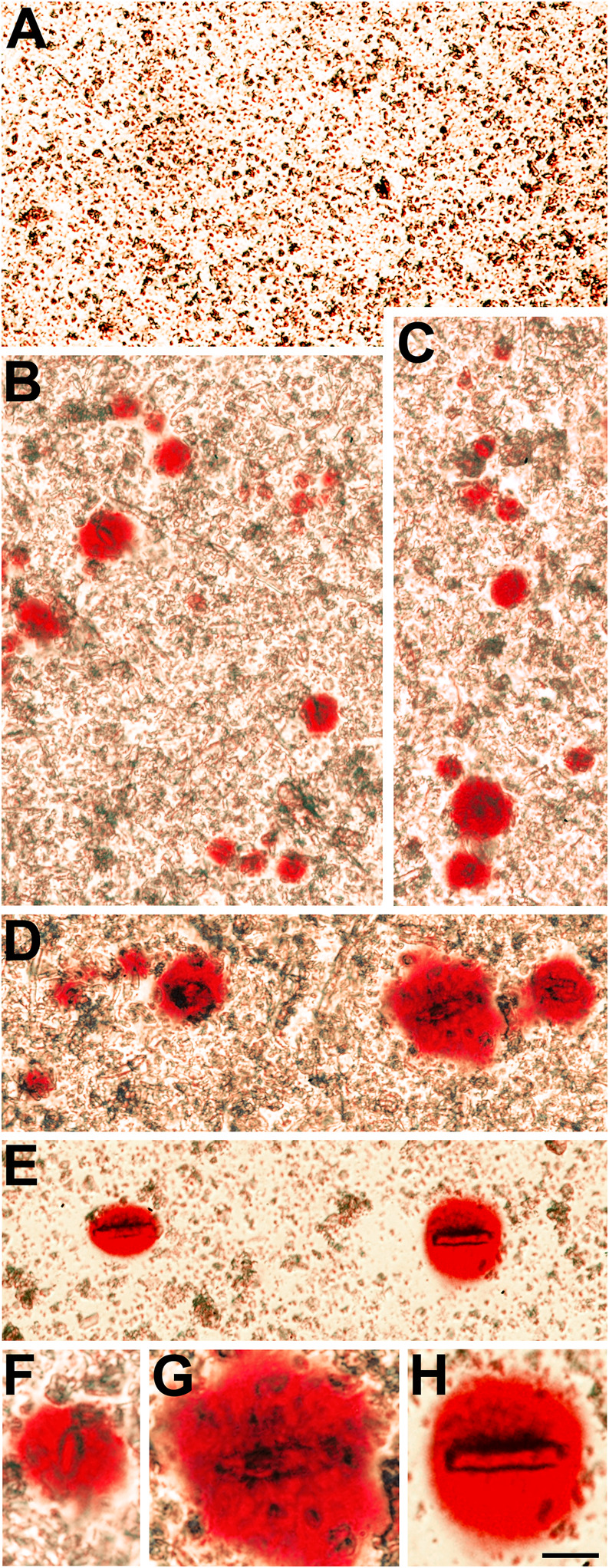
The insoluble fraction of homozygous Cx50D47A lenses contains Alizarin red-stained crystals. **(A)** Image shows the lack of Alizarin red-stained material in the lens insoluble fraction from a 93-day-old wild-type Cx50D47A female mouse. **(B−E)** Images show several Alizarin red-stained crystals present in the insoluble fraction of Cx50D47A homozygous lenses from a 60-day-old female mouse **(E)** and a 93-day-old male mouse **(B–D)**. **(F−H)** Higher magnification images of selected Alizarin red-stained crystals from panels **(B,D,E)**. The images were modified to decrease the reddish background from the Alizarin red solution used to resuspend the insoluble fraction. The scale bar represents 54 µm in panels **(A−E)**, and 27 µm in panels **(F−H)**.

We also sought to further characterize the Alizarin red-stained material that we had previously detected after wholemount staining of homozygous Cx50D47A and Cx46fs380 lenses (Gao et al., 2018; Berthoud et al., 2019). For this purpose, we prepared insoluble fractions from lens homogenates from wild-type and homozygous Cx50D47A and Cx46fs380 mice by centrifugation and stained them with Alizarin red. No significant stained material was detected in the insoluble fraction from wild-type lenses of either mouse line (Figures 3A, 4A). In contrast, the samples from homozygous lenses of both lines contained particles that stained with Alizarin red (Figures 3, 4). In the homogenates of the Cx46fs380 lenses, the Alizarin red-stained objects varied widely in size, but the size range was within the dimensions determined by micro-CT (i.e., 22–310 µm in the longest dimension for the examples shown in Figure 3). Many of the particles in the Cx46fs380 insoluble fractions had distinct, sharp edges. In the homogenates from the Cx50D47A lenses, most of the Alizarin red-stained particles appeared as irregularly shaped red objects of various sizes (ranging from <8 to 70 µm in the longest dimension for the examples shown in Figure 4). Some of the particles had dark, sharp, linear edges suggestive of` crystals (shown at higher magnification in Figures 4F−H).

### Identification of the Lens Mineral

**FIGURE 5:**
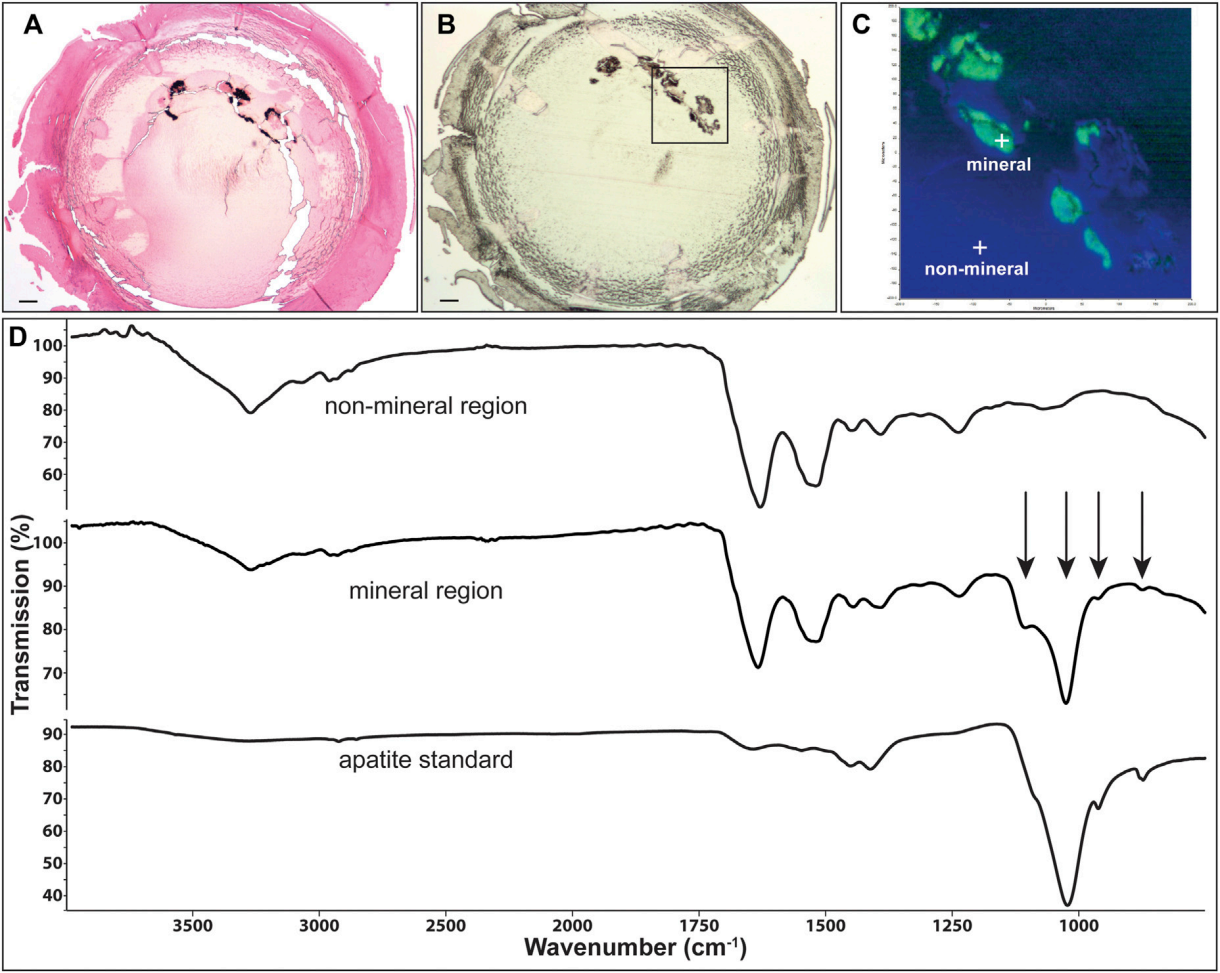
Identification of mineral type in Cx46fs380 lenses. **(A)** Image showing Yasue staining of a section from the lens of the 171-day old homozygous Cx46fs380 male mouse shown in Figure 1. The black stained material corresponds to calcifications. **(B)** Image of an unstained section of the same lens on low emissivity glass that was used for infrared imaging. **(C)** Image shows the infrared signal map obtained of the boxed region shown on **(B)** by collecting 65,746 infrared spectra with a pixel resolution of 1.56 μm. Green areas are mineral regions and blue areas are protein regions. **(D)** Graphs show the spectra of a non-mineral region, a mineral region, and the apatite standard. Arrows point to features characteristic of apatite mineral in the lens. The scale bars in **(A,B)** represent 100 μm.

**FIGURE 6:**
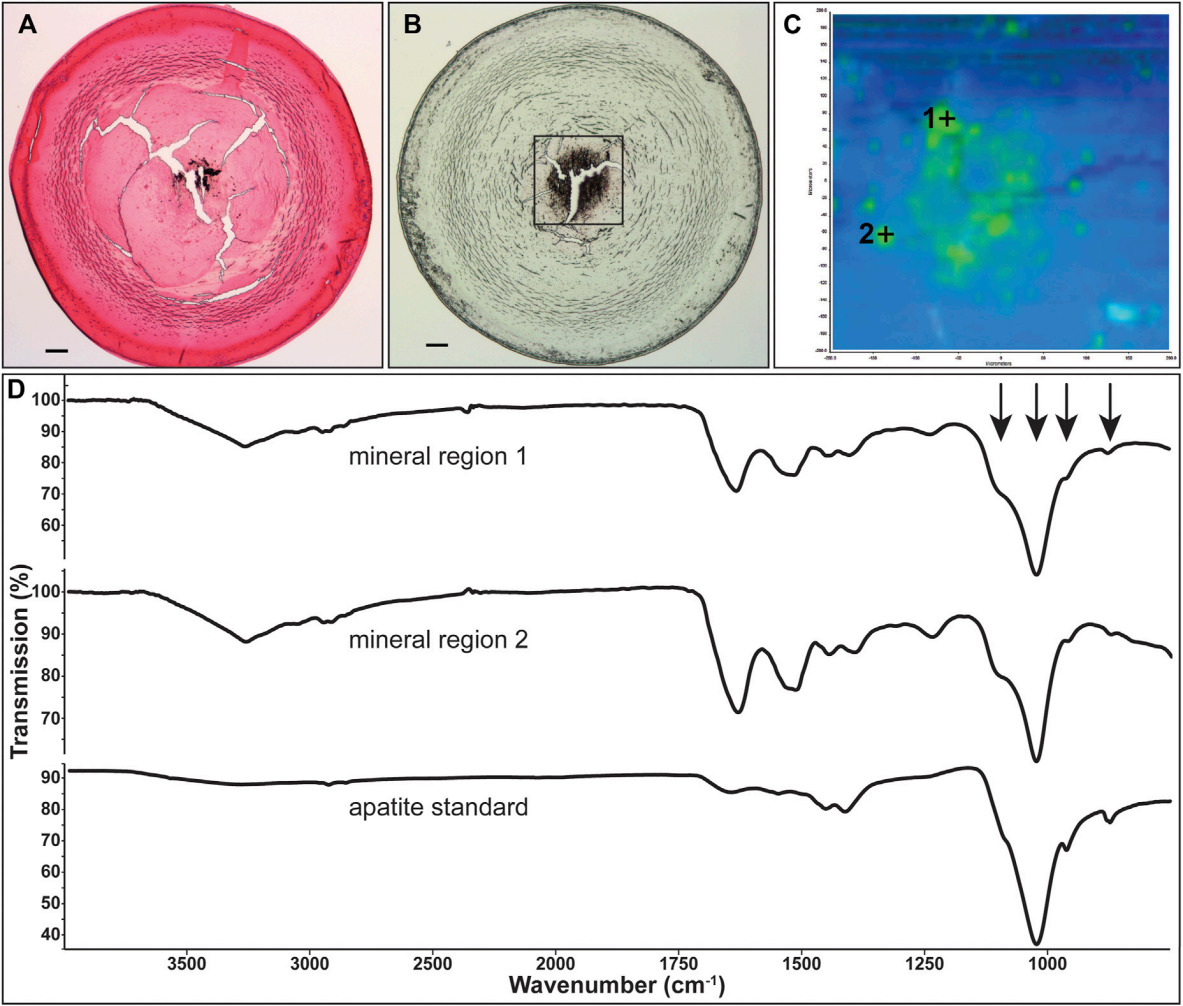
Identification of mineral type in Cx50D47A lenses. **(A)** Image showing Yasue staining of a section from the lens of the 102-day-old homozygous Cx50D47A male mouse shown in Figure 2E. The stained material that appears black corresponds to calcifications. **(B)** Image of an unstained section of the same lens on low emissivity glass used for infrared imaging. **(C)** Image shows the infrared signal map of the boxed region shown on **(B)** generated by collecting 4,096 infrared spectra with a pixel resolution of 6.24 μm. Green areas are mineral regions and blue areas are protein regions. **(D)** Graphs show the spectra of two mineral regions and the apatite standard. Arrows point to features characteristic of apatite mineral in the lens. The scale bars in **(A,B)** represent 100 µm.

To determine the composition of the mineral particles, sections from homozygous Cx46fs380 or Cx50D47A lenses were mounted on low-E glass slides. These sections were then analyzed using Attenuated Total Internal Reflection micro–Fourier transform infrared spectroscopy (ATR-μFTIR) imaging. Localization of Ca^2+^-containing material in Yasue-stained sections facilitated identification of regions in adjacent sections to analyze by ATR-μFTIR (Figures 5A−C and Figures 6A−C). Sections from the lenses of at least three homozygous Cx46fs380 and Cx50D47A mice were analyzed using this technique.

Representative spectra extracted from the non-mineral regions and the mineral-rich regions in sections of homozygous Cx46fs380 lenses are shown in Figure 5D. The spectra extracted from the non-mineral regions are characteristic for normal protein. Prominent features in these spectra include the combined N-H and O-H stretch located near 3,270 cm^−1^ and the amide I and II absorptions located near 1,633 and 1,521 cm^−1^, respectively. The spectra extracted from the mineral-rich regions showed additional absorptions besides those observed in spectra from the non-mineral regions. These additional absorptions are located at 1,106, 1,024, 961, and 873 cm^−1^, which are characteristic for calcium apatite. A reference spectrum of apatite is also shown in Figure 5D for comparison. Similarly, in sections of homozygous Cx50D47A lenses the infrared spectra of the mineral regions showed the absorption bands characteristic of protein and of calcium apatite (Figure 6D).

Thus, these analyses revealed that the composition of the mineral present in both Cx46fs380 and Cx50D47A lenses contains calcium phosphate in the form of apatite.

## Discussion

In this manuscript, we have demonstrated that cataracts resulting from mutations in the lens fiber cell connexins, Cx46 and Cx50, form by deposition of calcium phosphate. We found insoluble, calcium-containing material in homogenates and X-ray dense material only in samples from cataractous lenses. In addition, in both mutant mouse lines, we identified the mineral component as apatite.

The current data from micro-computed tomographic scans show that the distributions of radio-dense material (mineral) closely correspond to the cataract morphologies in both mouse models. In addition, the three-dimensional distribution of the X-ray dense material observed by micro-CT scanning of cataractous lenses from Cx46fs380 and Cx50D47A mice closely resembles the Alizarin red staining pattern of these lenses (Gao et al., 2018; Berthoud et al., 2019). Thus, the micro-CT scanning data provide conclusive evidence that cataracts reflect the formation of calcified deposits.

ATR-μFTIR allowed us to study the chemical composition of the cataracts in the Cx46fs380 and Cx50D47A mice. The ATR-μFTIR spectral data show that the inorganic component of the mineralized particles in the lenses of both mouse lines is calcium phosphate (apatite). This mineral form of calcium is often found in other body tissues/fluids where calcium deposits have been reported. Typically, apatite forms acicular crystallites (∼0.1 µm in length) that are closely bound with proteins (LeGeros, 2001; Ryall, 2008). Our ATR-μFTIR spectral data also showed amide I and amide II bands at the sampled locations. The spatial resolution of the method is diffraction limited to 6 μm at 1,650 cm^−1^ and to ∼3 μm at 3,400 cm^−1^ in the x-y plane. In the z plane, the infrared beam penetrates the sample to depths of 0.67 and 0.33 μm at those wavenumbers, respectively, but the penetration depth into the sample in the ATR method increases from the short wavelength (high wavenumber) to the long wavelength (low wavenumber) end of the spectrum (e.g., 1.1 μm at 1,000 cm^−1^). Therefore, based on the large size of many of the particles relative to the spatial limitations of the method and the avidity of apatite crystallites for protein, it is likely that proteins are associated with the mineral. These proteins may be integral components of the mineral, but it is also possible that the proteins surround the crystals or are trapped inside them during their formation. The detection of both a calcium salt and protein at locations of mineral deposits is consistent with the deposition of crystals on a macromolecular matrix as occurs during physiological biomineralization of tissues like bone and teeth. Since the formation of calcium depositions within the lens corresponds to a pathological phenomenon that is detrimental to lens function, we consider this process to be “pathological mineralization.”

**FIGURE 7:**
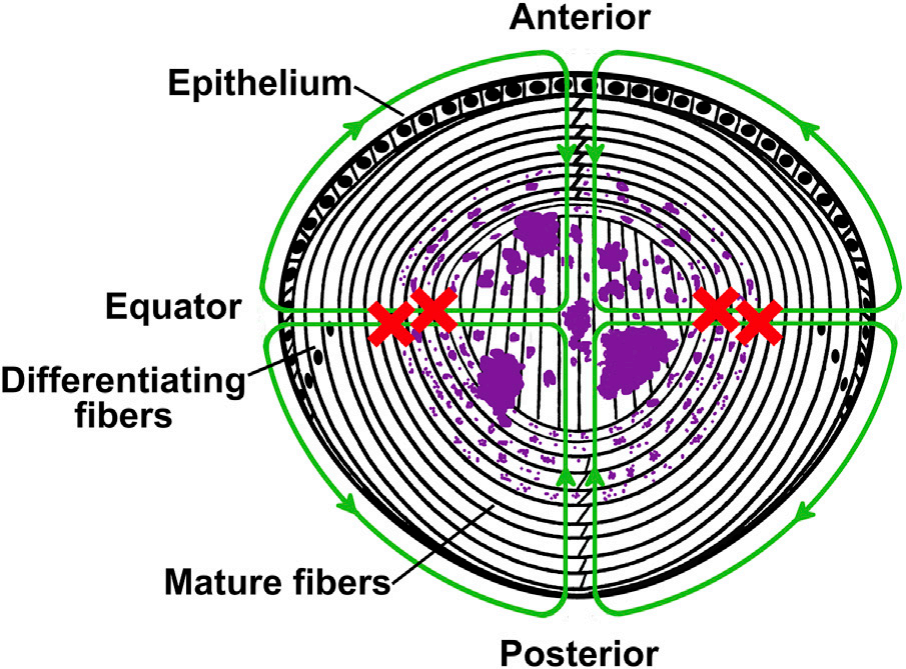
Model illustrating the mechanism of formation of apatite deposits in the lens. The diagram depicts a cross sectional view of a mouse lens in which the lens circulation is impaired due to expression of a mutant connexin in the fiber cells. In the healthy lens, ions and fluid enter into the lens from the anterior and posterior poles and move to the lens center through the extracellular spaces, and they exit across epithelial cell membranes at the equator. The green lines with arrowheads illustrate the circulation and the direction of flow of ions and fluid in a normal healthy lens. The red crosses indicate that outflow of ions through fiber cell-to-fiber cell gap junctions is impaired due to the expression of the mutant connexin. This disruption leads to accumulation of calcium ions. As their concentration increases, the calcium ions react with the intracellular phosphate to form apatite precipitates (illustrated in purple). The crystals are initially small, but they become larger with aging.

Precipitation of calcium salts requires supersaturating concentrations of calcium ions (and an anion). We have previously shown that the Cx46fs380 and Cx50D47A lenses contain increased levels of intracellular calcium ions and that these levels surpass the *Ksp* for some of its salts (Gao et al., 2018; Berthoud et al., 2019). We have developed a model to explain the mechanism of formation of the calcium precipitates in connexin mutant lenses that is presented in Figure 7. The lens microcirculation is impaired in homozygous connexin mutant lenses, because gap junctional intercellular communication, which provides the outflow pathway for ions to exit the lens, is significantly decreased (Minogue et al., 2017; Berthoud et al., 2019). Consequently, ions accumulate in the lens. In the case of calcium ions, the intracellular concentration of free calcium ions reaches values above 2 μM in the center of the lens in both fiber cell connexin mutant lenses, whereas in surface fiber cells it is similar to wild-type in homozygous Cx46fs380 lenses and more than twice the value in wild-type lenses in homozygous Cx50D47A lenses (Gao et al., 2018; Berthoud et al., 2019). The high intracellular concentration of calcium ions reacts with intracellular inorganic free phosphate to form calcium phosphate (apatite).

Several lines of evidence suggest that an increase in the concentration of calcium to supersaturating levels followed by formation of calcium deposits is a common and necessary step in the formation of lens opacities of many different etiologies. Increased levels of Ca^2+^ have been reported in human and mouse lenses with cataracts of various etiologies (Duncan and van Heyningen, 1977; Hightower and Reddy, 1982; Duncan and Jacob, 1984; Gao et al., 2004; Li et al., 2010; Liu et al., 2015). Indeed, many years ago cataracts were reported to contain insoluble material that appeared like scales or needles (which were suggested to be composed of different substances, including phosphate of lime (a term used then for calcium phosphate) and cholesterine) (reviewed and discussed in Jacob, 1851). Subsequent studies have reported increased levels of calcium ions in the insoluble fraction of human cataractous lenses compared with non-cataractous lenses (Duncan and van Heyningen, 1977), X-ray dense material and calcium-rich regions in the cortical region of canine cataractous lenses (Antunes et al., 2006), and radio-dense material accumulated in the anterior Y suture of Cx46-null mice (Li et al., 2021). Similar to our previous studies of lens fiber cell connexin mutant mice (Gao et al., 2018; Berthoud et al., 2019), material that stains with Alizarin red has been detected in cataractous lenses from mice carrying a mutation in the transient-receptor-potential cation channel, subfamily M, member 3 (TRPM3) and in Cx46-null mice (Li et al., 2021; Zhou et al., 2021).

Our study provides evidence for cataractogenesis through formation of insoluble, calcified material in which the counterion is phosphate. Similar to our findings in connexin mutant lenses, several case reports have identified mineral composed of calcium and phosphate in the cataracts of both younger and older people (Fagerholm et al., 1982; Fagerholm et al., 1986; Chen et al., 2005). Although calcium precipitates may be a common finding in cataracts of different etiologies, the counterion in the precipitates might differ as the presence of calcium oxalate has been reported in Morgagnian cataracts (Zimmerman and Johnson, 1958; Bron and Habgood, 1976; Pau, 1984). Nevertheless, the presence of apatite or another precipitated calcium salt in the lens (whether deposited so densely that it effectively blocks X-rays or deposited less abundantly so that it causes a much lower total X-ray attenuation (Williams et al., 2021)) would alter the gradient of refractive index and cause density fluctuations and light scattering.

Thus, our current data suggest that the formation of cataracts in two different mouse models occurs through pathological mineralization of the organ, and consideration of published reports implies that this process may be a general mechanism contributing to the formation of cataracts of many different etiologies.

## Materials and Methods

### Animals

Cx46fs380 animals were generated and maintained as described by Berthoud et al. (2014). Cx50D47A mice (also known as *No2* or ENU-326) (Favor, 1983) were maintained in the C3H mouse strain as described previously (Berthoud et al., 2013). Animals of both sexes were used for all experiment types. The sex of the animal is indicated in the legends for the data shown in the figures. Cx46fs380 animals were studied at 157, 167, 171 and 191 days. Cx50D47A animals were studied at 56, 59, 60, 63, 93 and 102 days. All animal procedures were approved by the University of Chicago Animal Care and Use Committee and followed its guidelines.

### Light Microscopy Analysis

Before fixation, lenses were viewed using a Zeiss Stemi-2000C stereo microscope (Carl Zeiss, München, Germany) equipped with a halogen lighting system for transmitted illumination. Images were acquired with a Zeiss AxioCam digital camera using Zeiss AxioVision software using identical settings (i.e., magnification, illumination, and exposure time) to photograph the wild-type and homozygous lenses in each group (Berthoud et al., 2013; Berthoud et al., 2014).

### High Resolution Micro-CT

Lenses were fixed in 5% paraformaldehyde in PBS for 48 h and scanned by high resolution micro-CT to detect the presence or absence of high X-ray attenuating (mineralized) material using a Skyscan 1172 Micro CT System at 60 kV with a final image stack resolution of 3–6 µm cubic voxels. We examined a total of 6 wild-type and 7 homozygous Cx46fs380 mouse lenses (157–171 days of age), and 14 wild-type and 14 homozygous Cx50D47A mouse lenses (56–102 days of age). The micro-CT scans were performed under blinded conditions by an investigator who was unaware of the genotypes. Three-dimensional image stacks were viewed using ImageJ (as distributed through fiji. sc) (Schindelin et al., 2012), and overall mineral density and distributions of deposit sizes were quantified. Movies from the three-dimensional scan stacks were generated using 3D Slicer (Fedorov et al., 2012).

### Alizarin Red Staining of the Lens Water-Insoluble Fraction

Wild-type and homozygous Cx46fs380 lenses from 191-day-old mice and wild-type and homozygous Cx50D47A lenses from 60 and 93 days of age were homogenized in PBS containing cOmplete EDTA-free protease inhibitor cocktail (Roche Applied Science, Indianapolis, IN, United States) at a concentration of 1 tablet/7 ml using a glass-glass homogenizer. The homogenates were centrifuged at 16,000 g for 20 min. The supernatant was discarded, and the pellet was resuspended in homogenization buffer. Equal amounts of the resuspended pellet and a filtered solution of 2% Alizarin red in water pH 4.1–4.3 were added to a glass slide and mixed by pipetting up and down. Glass slides and the glass-glass homogenizer were pretested to make sure they did not contain any material that interacted with Alizarin red and cleaned with deionized water prior to the experiment. The specimens were observed using a DIAPHOT inverted Nikon microscope (Nikon Instruments Inc., Melville, NY) equipped with epifluorescence and Hoffman modulation contrast optics. Phase-contrast and fluorescence images were obtained with a 10X objective using a Nikon D70 digital camera (Nikon). These experiments were performed once for Cx46fs380 lenses and twice for Cx50D47A lenses. The longest dimension of the Alizarin red-stained particles was determined using ImageJ (Schindelin et al., 2012).

### Yasue Staining

After micro-CT scanning, each lens was dehydrated and embedded in paraffin. Five-μm sections were obtained from each lens, and alternating sections were deposited on regular glass for staining with the Yasue method to show calcium salts (Yasue, 1969) and on low-emissivity (low-E) glass for infrared microspectroscopy.

### Fourier-Transform Infrared Microspectroscopy

The composition of crystals in Cx46fs380 lenses (157–171 days of age) and Cx50D47A lenses (102 days of age) was determined by Attenuated Total Internal Reflection Fourier-transform infrared microspectroscopy (ATR-μFTIR) on lens sections mounted on low-E glass slides. The unstained tissue sections were imaged using the visible CCD camera and frame grabber on the Spectrum Spotlight (Gulley-Stahl et al., 2010). Infrared images over the selected area were collected with a Perkin-Elmer Spotlight 400 infrared microscope interfaced to a Perkin-Elmer Frontier Fourier transform infrared spectrometer (FTIR). The system employed a 16 × 1, liquid nitrogen cooled, mercury cadmium telluride (HgCdTe) array detector. The ATR imaging accessory is based on a germanium internal reflection element which enables infrared spectra to be collected at a pixel resolution of 1.56 μm or 6.24 μm. Each spectrum in the image represents the average of four individual scans collected at a spectral resolution of 8 wavenumbers (cm^−1^).

### Statistics

Data are presented as a range or mean (with its range in the case of percentages). Statistical analysis of the width and length of X-ray dense material was performed using unpaired two-tailed Student’s t-test. A *p* value < 0.05 was considered significant. Data from both sexes were combined because no sex differences were detected in the parameters studied.

## Data Availability

The original contributions presented in the study are included in the article/Supplementary Material, further inquiries can be directed to the corresponding author.

## Supplementary Material

Supplementary Movie S1: Animation of the series of scans through the lens of a 157-day-old homozygous Cx46fs380 male mouse. In this animation, the lens has been oriented with its anterior face towards the bottom.

Supplementary Movie S2: Animation of the series of scans through the lens from a 56-day-old homozygous Cx50D47A male mouse shown in Figure 2B.

Supplementary Movie S3: Animation of the series of scans through the lens from a 102-day-old homozygous Cx50D47A male mouse shown in Figure 2E.

## Abbreviations

ATR-μFTIR, attenuated total internal reflection micro–Fourier transform infrared spectroscopy; Cx46, connexin46; Cx50, connexin50; micro-CT, micro-computed tomography.
